# The Diagnosis of Small-Pox

**Published:** 1901-10-12

**Authors:** 


### Hospital Clinics and Medical Progress.

### THE DIAGNOSIS OF SMALL-POX.

The difficulties of diagnosis which surround many a case of small-pox in its early stages are by no means to be made light of. No disease declares its nature more markedly than a well-developed case of typical small-pox, but even in such a case it is easy to make mistakes in the early stages, while in cases of modified small-pox it may be quite impossible to come to a definite conclusion from the phenomena presented by a single case standing apart from its history. Let us take first the difficulties which exist in the early stage of the disease. At this period the initial erythema is apt to lead to error. When we have to do with a case in which there are headache, vomiting, pyrexia and a rash, the question may arise whether it is one of the other eruptive fevers, for the initial rashes of small-pox sometimes show considerable resemblance to the rashes of measles, or scarlet fever. When the initial erythema of small-pox is general it may be almost impossible to distinguish it from scarlet fever, but on carefully examining it, it will usually be found, according to some authorities, that it does not present that punctate appearance which, when the rash of scarlet fever is bright and widely diffused, may almost always be seen in parts. Other authorities hold that the initial rash of small-pox has just the same punctate appearance as that of scarlet fever. It is not often, however, so wide spread as to lead to error, the erythema of small-pox being usually limited in extent. Again, in scarlet fever so widely spread there will surely be some affection of the fauces and soft palate, with swelling of the tonsils and the cervical glands; while if the eruption be only partial, it will probably not occupy the parts most favoured in scarlet fever, namely, on the neck and upper part of the chest. Lastly, an important point to remember is that the initial scarletiniform rashes of small-pox do not occur in children under about 10 years of age, the time of life when scarlet fever is most common. In regard to the question of measles, one must not forget the preliminary catarrh which almost invariably precedes the measles rash. In small-pox the initial rash occurs much earlier in the illness than is the case in measles, and it comes out on the face, trunk, and extremities simultaneously, whereas in measles it begins behind the ears and on the forehead, chin, cheeks and neck, and then spreads to the rest of the surface. Putting initial rashes on one side, there are two symptoms by which we may easily be misled, unless by the pulse and the temperature our attention is directed to the fact that we have to do with an acute febrile disorder, namely, vomiting and backache. Cases occur in which the vomiting is so prominent a symptom and one so suggestive of stomach disorder, as to lead to mere symptomatic treatment, while in other cases the severity of the backache may lead to a diagnosis of lumbago. When the proper rash of small-pox first makes its appearance it may be mistaken for many things, and again in this stage measles is a disease with which it is often confused. It is the confluent form of small-pox which on the first day or two of the rash is so often mistaken for measles. The face and arms in such a case may be congested and of a purplish-red colour, very like that which is sometimes seen in bad cases of measles, and studded with slightly raised papules not entirely unlike the eruption in that disease; moreover, from the puffiness of the eyelids and the suffusion of the eyes, the resemblance to measles may be still further increased. If the case be one of small-pox, however, it will be found that the papules on the forehead are more raised and resistent, more approaching to "shottiness" than is ever the case with measles, while on some of the papules small vesicles are forming, and on examining the mouth and soft palate small white vesicular spots may be found on the mucous membrane.

While measles is the disease with which the confluent form of small-pox is perhaps most often mistaken, chicken-pox is that with which confusion mostly arises in the discrete form. There ought, however, to be no mistake if the case be seen on the first or second day of the eruption, and if one can trust to the history given. The lack of any marked rise of temperature; the distribution of the chicken-pox eruption, which is most abundant on the trunk or on the scalp, while it is much less so on the face, and still less on the hands and feet; the manner in which it comes out in successive crops, and in which each crop rapidly matures, becoming vesicular within a few hours of its appearance; the frequently oval shape of the vesicles on the abdomen, chest, and back, all point to chicken-pox. While, on the other hand, the marked initial symptoms with high fever; the fact that the eruption does not become vesicular within a few, say twelve, hours of its appearance, and that, when formed, the vesicles are not oval, and do not collapse on being pricked, as the chicken-pox vesicle does; and finally that the temperature falls with the outcoming of the eruption, which is most marked on the face and extremities, all point to smallpox. When, however, the eruption has been scratched, and the febrile period is over, it may be impossible to say whether the disease has been chicken-pox or modified smallpox. The distribution of the rash and the history, if given by an observant person, may, however, be very suggestive. One point in the history one should always inquire about and give due emphasis to, namely, the previous existence of small-pox in the house. We venture to say that, however sure one may feel about a case being chicken-pox, one should hesitate in pronouncing it as such if there be a history of a recent case of small-pox in the same house, while, if the rash is in the slightest degree doubtful, such a history should lead us at once to deal with the case as one of the major disease. It is curious that in the course of most small-pox epidemics errors arise in regard to the diagnosis between this disease and syphilis. Such mistakes generally occur with regard to the papular and pustular forms of syphilis, and from overlooking the fact that a widespread manifestation of this disease is sometimes accompanied with a certain degree of pyrexia. It will generally be found that however accurately some of the spots simulate smallpox, a variety of other forms of syphilitic rash will be found in other parts; in this as in other instances the polymorphism of syphilis being the key to the diagnosis. Moreover, the pyrexia, such as it is, does not subside, as that of small-pox does, on the develop ment of the rash. It must be confessed, however, on the other hand, that an attack of modified small-pox in a syphilitic subject is very liable to be overlooked. One would hardly think that mistakes would occur in regard to the eruption of typhoid fever. In yet such is the case. In some cases a typhoid eruption may be distinctly prominent, and although the length of time that the disease has existed ought to put all question of small-pox out of court, we have to remember that some cases of typhoid may be walking about far into the disease, so that on the breakdown occurring we may find a high temperature ; spots, prominent and tipped with minute vesicles ; and a history of only two or three days of illness ; so that a mistake is very possible if an immediate diagnosis is demanded. Many other eruptions—if only a small part of the surface be inspected—may strongly suggest smallpox, and the only way to escape error is, on the one hand, to inspect the whole surface of the body, and, on the other hand, to carefully compare the date and the apparent stage of the disease with the condition of eruption which is discovered. Time, of course, clears up all things, and among them most questions of diagnosis. But in the diagnosis of smallpox time is not allowed us ; and if for the benefit of the public rapid diagnosis is insisted on, so that all affected persons may be quickly isolated, mistakes are sure now and again to occur, even with the greatest care.

